# A complex rearrangement between *APC* and *TP63* associated with familial adenomatous polyposis identified by multimodal genomic analysis: a case report

**DOI:** 10.3389/fonc.2023.1205847

**Published:** 2023-08-03

**Authors:** Satoyo Oda, Mineko Ushiama, Wataru Nakamura, Masahiro Gotoh, Noriko Tanabe, Tomoko Watanabe, Yoko Odaka, Kazuhiko Aoyagi, Hiromi Sakamoto, Takeshi Nakajima, Kokichi Sugano, Teruhiko Yoshida, Yuichi Shiraishi, Makoto Hirata

**Affiliations:** ^1^ Department of Genetic Medicine and Services, National Cancer Center Hospital, Tokyo, Japan; ^2^ Department of Clinical Laboratories, National Cancer Center Hospital, Tokyo, Japan; ^3^ Department of Clinical Genomics, National Cancer Center Research Institute, Tokyo, Japan; ^4^ Division of Genome Analysis Platform Development, National Cancer Center Research Institute, Tokyo, Japan; ^5^ Department Medical Ethics/Medical Genetics, Kyoto University Graduate School of Medicine, Kyoto, Japan; ^6^ Department of Clinical Genetics, Cancer Institute Hospital, Japanese Foundation for Cancer Research, Tokyo, Japan; ^7^ Department of Genetic Medicine, Kyoundo Hospital, Sasaki Foundation, Tokyo, Japan; ^8^ Division of Molecular Pathology, National Cancer Center Research Institute, Tokyo, Japan

**Keywords:** familial adenomatous polyposis, APC regulator of WNT signaling pathway, tumor protein 63, multi-gene panel testing, adaptive sampling

## Abstract

Genetic testing of the *APC* gene by sequencing analysis and MLPA is available across commercial laboratories for the definitive genetic diagnosis of familial adenomatous polyposis (FAP). However, some genetic alterations are difficult to detect using conventional analyses. Here, we report a case of a complex genomic *APC-TP63* rearrangement, which was identified in a patient with FAP by a series of genomic analyses, including multigene panel testing, chromosomal analyses, and long-read sequencing. A woman in her thirties was diagnosed with FAP due to multiple polyps in her colon and underwent total colectomy. Subsequent examination revealed fundic gland polyposis. No family history suggesting FAP was noted except for a first-degree relative with desmoid fibromatosis. The conventional *APC* gene testing was performed by her former doctor, but no pathogenic variant was detected, except for 2 variants of unknown significance. The patient was referred to our hospital for further genetic analysis. After obtaining informed consent in genetic counseling, we conducted a multigene panel analysis. As insertion of a part of the *TP63* sequence was detected within exon16 of *APC*, further analyses, including chromosomal analysis and long-read sequencing, were performed and a complex translocation between chromosomes 3 and 5 containing several breakpoints in *TP63* and *APC* was identified. No phenotype associated with *TP63* pathogenic variants, such as split-hand/foot malformation (SHFM) or ectrodactyly, ectodermal dysplasia, or cleft lip/palate syndrome (EEC) was identified in the patient or her relatives. Multimodal genomic analyses should be considered in cases where no pathogenic germline variants are detected by conventional genetic testing despite an evident medical or family history of hereditary cancer syndromes.

## Introduction

Familial adenomatous polyposis (FAP, OMIM: #175100) is a rare colorectal cancer predisposition syndrome that is characterized by the occurrence of multiple adenomatous colonic polyps. This condition is also associated with various clinical features, such as gastric fundic gland polyposis, desmoid fibromatosis, adenoma of the ampulla of Vater, hepatoblastoma, and thyroid cancer (1). As FAP is caused by germline pathogenic variants of the APC regulator of the WNT signaling pathway (*APC*) gene, genotyping of the *APC* gene is often essential for determining the clinical management of patients, based on the reported genotype-phenotype correlations (1–6). Currently, *APC* molecular genetic testing, including coding sequence analysis and gene-targeted deletion or duplication analysis, is available across commercial laboratories in Japan. However, such conventional analyses occasionally fail to detect pathogenic variants in a subset of patients with an evident phenotype and family history of FAP (7–15). This is partly because conventional analytic methods hardly detect some types of genomic alterations, such as large-scale or complex structural variants or variants located in noncoding regions, including deep introns, and insertions or deletions of repetitive elements. Long-read sequencing has recently attracted attention as a novel analytic method that compensates for such weaknesses of conventional sequencing (16).

This study reports a case with a complex *APC-TP63* rearrangement in a patient with FAP that was not identified by conventional genetic analysis but by a series of genetic analyses including multigene panel testing, chromosomal analysis, and long-read sequencing.

## Case presentation

The patient was a woman in her thirties. Because of a positive fecal occult blood test result at an annual colorectal screening test, she consulted another hospital and was diagnosed with FAP due to multiple polyps (approximately 300 adenomas) in her colon. She underwent total colectomy and subsequent gastrointestinal examination, which revealed fundic gland polyposis. No other overt phenotypes or anomalies were identified. One of her first-degree relatives, her child had also exhibited desmoid fibromatosis, which is a rare disease associated with FAP, in his/her pre-teen years, while he/she did not have a colonoscopy because he/she was not yet at high risk of polyposis. No associated disease or phenotype was identified in other relatives including the proband’s parents (**Supplementary Table S1**). *APC* gene testing, including targeted next-generation sequencing and MLPA was submitted to a commercial laboratory by her former attending doctor, but with the exception of 2 variants of unknown significance, no pathogenic variant was detected in *APC*. The patient was then referred to our hospital for further genetic analysis. After obtaining informed consent for comprehensive genomic analysis of cancer predisposition syndromes, we first conducted a multigene panel (MGP) analysis.

For MGP testing, we used SureSelect Custom DNA Target Enrichment Probes (Agilent, Santa Clara, CA), which were originally designed to target 147 cancer-predisposing genes, including *APC* and other genes associated with colorectal cancer or polyposis. The target regions cover exons, intronic sequences at the exon-intron junctions, promoters, and some other sequences in the introns of high-penetrance genes. Subsequently, sequencing and data analysis was performed using NextSeq (Illumina, San Diego, CA) and csDAI ver3.0 (Mizuho Res. & Technol., Tokyo, Japan).

**Figure 1:**
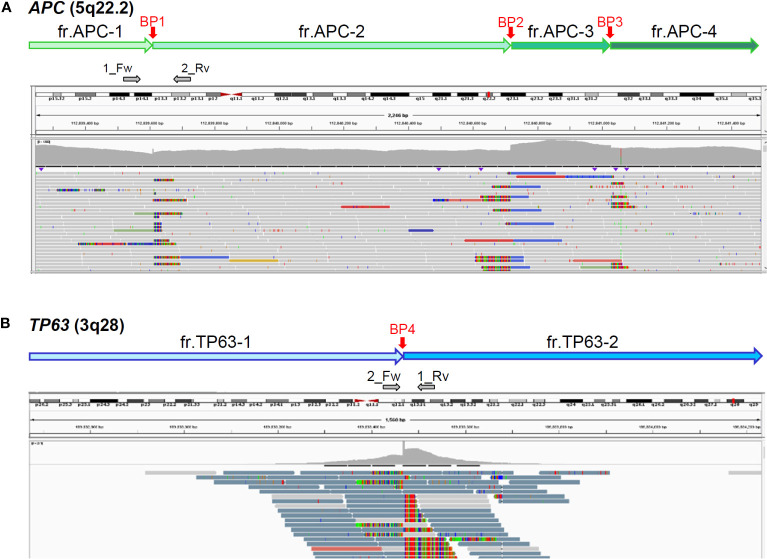
Presumed breakpoints in *APC* **(A)** and *TP63* **(B)** by integrative genomics viewer (IGV). Top box of IGV indicates chromosomal positions; middle, mapped read depth; bottom, mapped read sequences. Vertical red arrows indicate 3 (BP1-3) and 1 (BP4) breakpoints in *APC* and *TP63*, respectively, which were presumed by soft-clipped reads and read-depth gaps. Horizontal grey arrows indicate the positions and directions of primers for Sanger sequencing (**Supplementary Table S2**).

**Figure 2:**
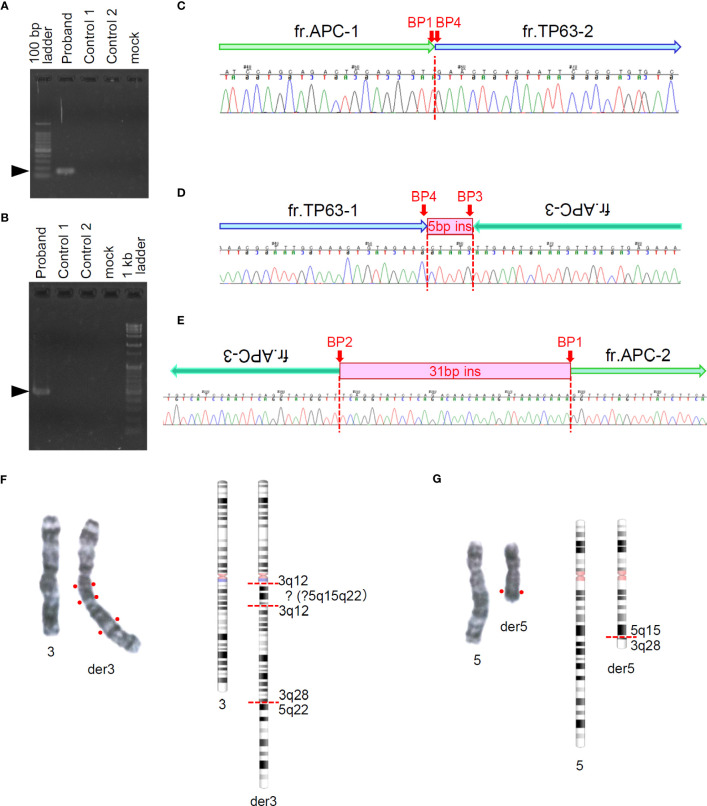
Sanger sequencing around presumed breakpoints in *APC* and *TP63* and G-banding of chromosomes 3 and 5. **(A, B)** Electrophoresis of amplicons of target regions for Sanger sequencing. Arrow heads indicate the target size (**A**: 155bp, **B**: 474bp) of PCR products. **(C–E)** Sequencing chromatograms around presumed breakpoints and schematics of rearranged genomic fragments. Fragment labels correspond to those shown in **Figures 1A and B**. **(F, G)** Right panels show G-banding images of paired chromosomes 3 **(F)** and 5 **(G)**. Left panels show G-banding images of paired chromosomes 3 **(F)** and 5 **(G)**. Right panels show chromosome schematics based on G-banding and spectral karyotyping (SKY) analyses.

As our analytic pipeline detected insertion of a part of the *TP63* sequence within exon16 of the *APC* gene, that is, APC : NM_000038.6:exon16:c.5433_5434insACAAAGGTTCTACTGTTTGCAAAGCGTTTTGCATTCTTTGGGTAAGAGGTGTTGGGCTTGTTATG(p.K1812Tfs*15), we reviewed the sequence read by IGV. Based on the soft-clipped sequences and gaps in read depth, we presumed the occurrence of 3 breakpoints in *APC* and 1 in *TP63* (**Figure 1**). Following a tentative definition of DNA fragments APC-1 to -4 and TP63-1 and -2 based on the IGV review, we presumed the occurrence of a rearrangement between *APC* and *TP63* and designed PCR primers for Sanger sequencing (**Figure 1** and **Supplementary Table S2**). Sanger sequencing was conducted using the BigDye Terminator v3.1 or v1.1 Cycle Sequencing Kit with an ABI PRISM 3130 Genetic Analyzer (Applied Biosystems, Foster City, CA). The SEQUENCHER v.5.4.6 (GENE CODES Corp., Ann Arbor, MI) was used to analyze and visualize the electropherograms. Sanger sequencing successfully validated each breakpoint and revealed a complex interchromosomal rearrangement between *APC* and *TP63* (**Figures 2A–E** and **Supplementary Figure S1**). We detected these breakpoint sequences in the proband and in a first-degree relative with desmoid fibromatosis. In addition, G-band and spectral karyotyping (SKY) at a commercial laboratory identified derivative chromosomes 3 (der[3]) and 5 (der[5]), exhibiting presumed interchromosomal rearrangements by MGP testing, as well as a novel insertion of 5q15-22 into 3q12 in der[3] and a rearrangement between 5q15 and 3q28 in der[5] (**Figures 2F, G** and **Supplementary Figure S2**), which were not identified in MGP testing and subsequent Sanger sequencing. To determine the loci of the other breakpoints detected by chromosomal analyses and validate the thorough rearrangements in chromosomes 3 and 5, we conducted long-read sequencing with adaptive sampling. For long-read sequencing, a DNA library was prepared using the SQK-LSK110 DNA ligation kit (Oxford Nanopore Technologies, Oxford, UK) and NEBNext (E7180S, New England Biolabos, Ipswich, MA) after shearing genomic DNA into approximately 20 kb fragment by G-TUBE (Covaris, Wobum, MA). The DNA library was subjected to GridION sequencing (Oxford Nanopore Technologies) with an adaptive sampling technique using R9.4.1 flow-cells (FLO-MIN106D, Oxford Nanopore Technologies), following the manufacturer’s protocol (16). Target regions for adaptive sampling were designed to cover the putative breakpoints of chromosomes 3 and 5 (**Supplementary Table S3**). Basecalling and fastq conversion were performed by MinKNOW ver. 22.10.7. We used Minimap2 for the alignment and generation of bam files (17) (mean coverage for the target regions: 25.5x), while Nanomonsv was employed for the detection of structural variants (18).

**Figure 3:**
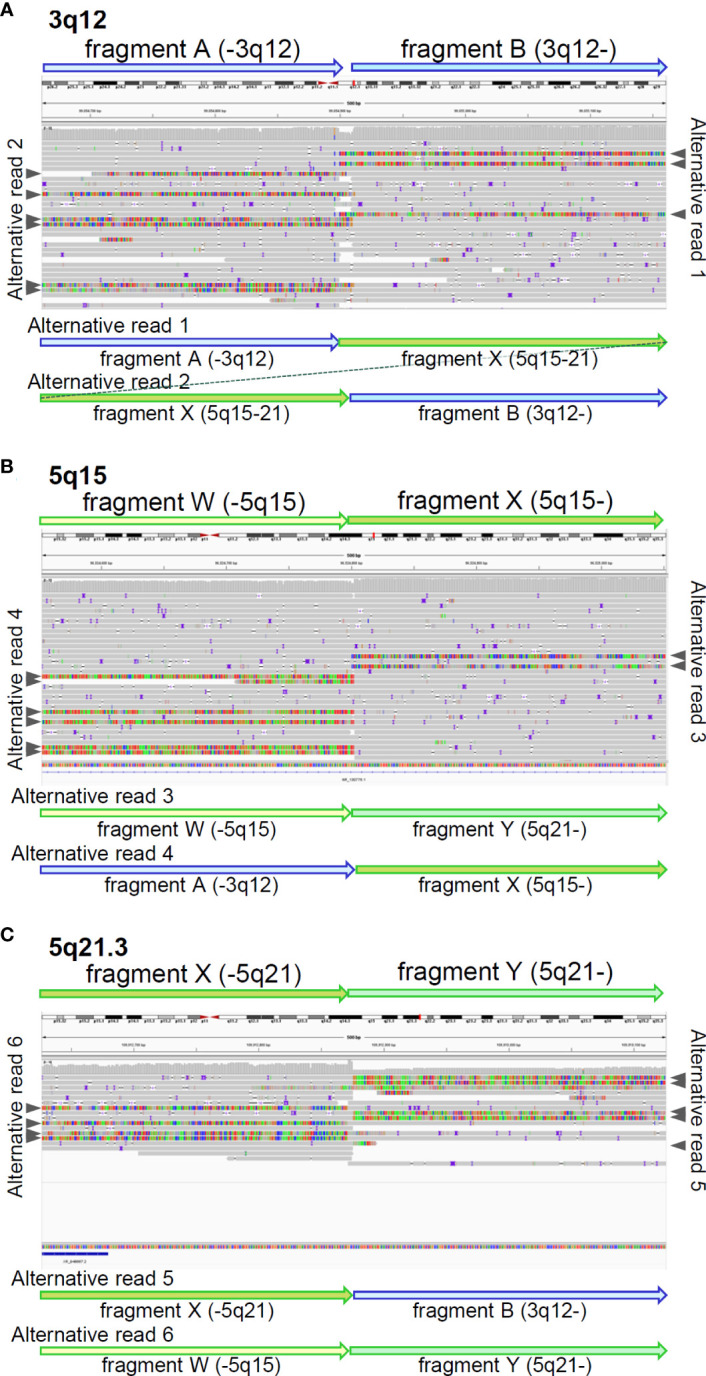
Breakpoints identified by long-read sequencing. IGV shows mapped reads with soft-clipped sequences around breakpoints on 3q12 **(A)**, 5q15 **(B)**, and 5q21.3 **(C)**. identified by long-read sequencing. Fragments were tentatively defined, based on the breakpoints. Grey arrow heads indicate soft-clipped alternative reads mapped in IGV. The soft-clipped alternative sequences were confirmed by BLAT and schematically displayed by fragment arrows.

**Figure 4:**
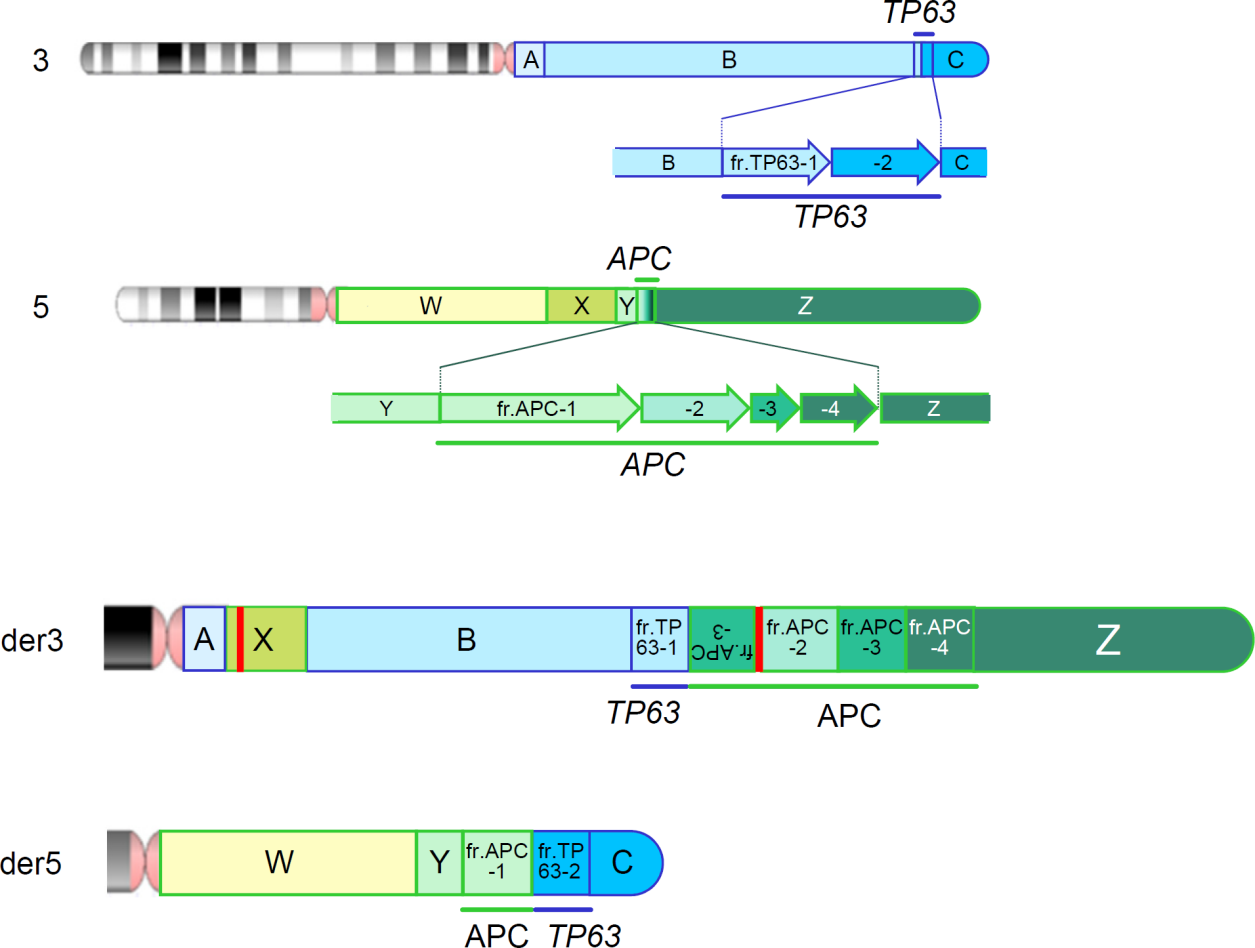
Chromosomal rearrangements identified by a series of genomic analyses. Upper 2 panels represent the wild-type chromosomes 3 and 5 and fragments tentatively defined by a series of genomic analysis. Bottom 2 panels (der3 and der5) represent the derivative chromosomes 3 and 5. Red vertical bars represent an additional insertion variant which is identified in fragment X (**Supplementary Figure S4**) and an insertion of unknown origin, which is identified within the structural variant of *APC*.

We also reviewed the genomic data using the integrative genomic viewer (IGV) ver. 2.13.1, with BAM files generated from the FASTQ files of MGP and long-read sequencing. Finally, long-read sequencing detected the precise breakpoints in chromosomes 3 and 5: insertion of chr5:96,524,803-109,912,875 (5q15-21) between chr3:99,054,892 and chr3:99,054,911 (3q12) in der[3], and deletion of chr5:96,524,798-109,912,872 (5q15-21) in der[5], which were identified as novel structural variants (**Figure 3** and **Supplementary Table S4**), in addition to the proven breakpoints in *APC* and *TP63* (**Supplementary Figure S3**). The *APC* structural variant was assumed to cause protein truncation of APC (p.Ser1340Ter) and to be a likely pathogenic variant. We identified another structural variant: an insertion of a simple-repeat region (**Supplementary Figure S4**, **Supplementary Table S4**), located in an intronic region of the Endoplasmic Reticulum Aminopeptidase 1 (*ERAP1*) gene. Finally, we present a schematic of the whole landscape of the structural variants of chromosomes 3 and 5 in **Figure 4** and **Supplementary Table S4**.

## Discussion

This study identified complex interchromosomal rearrangements between chromosomes 3 and 5 and their precise breakpoints using a series of integrated genomic analyses, including G-banding, Sanger sequencing, MGP analysis, and long-read sequencing (**Figure 4**).

Conventional genetic testing for *APC* includes sequence analysis that targets coding and splicing regions, and gene-targeted deletion/duplication analyses such as MLPA. However, such analyses occasionally fail to detect certain types of genetic variants. Previous genetic testing of *APC* has failed to identify pathogenic variants in 18–50% of patients with FAP (7–15). MGP analysis does not identify the whole landscape of genetic variants, but we included some of the deep-intronic regions of *APC* and other high-penetrant genes in the design of our MGP to aid copy number and other structural analyses in the computational analytical pipelines, which suggested chromosomal rearrangements in the patient. This finding prompted us to conduct further chromosomal analyses, such as G-banding and SKY, which detected interchromosomal rearrangements between chromosomes 3 and 5 with several breakpoints. We finally performed long-read sequencing using an adaptive sampling technique targeting putative breakpoint regions and identified each precise breakpoint on chromosomes 3 and 5 (**Figure 4** and **Supplementary Table S4**).

Long-read sequencing analysis is useful for the detection of complex chromosomal structural alterations. Recently, an adaptive sampling technique has been developed for efficient analysis of target regions of interest (16). In fact, detection of novel pathogenic variants using this novel analysis technique has been reported in various genetic diseases (19–21). To define the target regions, prior examinations using other genomic analyses, including MGP and chromosomal analysis, are essential, indicating the importance of integrated and cascade genomic analysis.

Importantly, a genotype-phenotype correlation of the *APC* gene has been shown in FAP. Pathogenic variants around the identified breakpoint, such as codon 1,340 in exon 16 of *APC* were located in the region, which has been associated with profuse FAP: codons 1,250-1,464 (1), while the occurrence of desmoid tumors has been associated with pathogenic variants in codons 400–1493 (4, 22). More than 100 but not profuse colorectal adenomatous polyps were found in the proband, while desmoid tumors were detected in her first relative, who also harbored the *APC* variant. As all these findings were not compatible with the genotype-phenotype correlation, more evidence regarding the genotype-phenotype correlation of *APC* rearrangements and its underlying mechanism needs to be accumulated to discuss the possibility of disease onset and suggest optimal surveillance.

Chromosomal rearrangements were also detected in the *TP63* gene. Because the breakpoint in *TP63* was in the common position of all transcript variants of *TP63* (23), the variant was presumed to be deleterious to the function of *TP63*. Pathogenic variants of *TP63* are known to be associated with split-hand/foot malformation (SHFM, OMIM: #605289) or ectrodactyly, ectodermal dysplasia, and cleft lip/palate syndrome (EEC, OMIM: #604292) (24). Moreover, homozygous deletion of *Trp63* in mice causes aplasia of limb buds (25). The proband and her relatives had no obvious phenotype associated with *TP63* pathogenic variants. Previous studies have shown that *TP63* missense variants might have a dominant negative effect on the function of *TP63* or frameshift variants might act as pathogenic affecting TP63-alpha isoforms (26–30). The variant identified in this study was detected in a heterozygous state, presumably inducing nonsense-mediated mRNA decay of all TP63 isoforms, which might not cause SHFM or EEC. However, as *TP63* is known to have a redundant function to that of *TP53* (31), pathogenic variants of which cause the Li-Fraumeni syndrome, we may need to consider a possibly higher risk of cancer and carefully follow up the patients with this variant.

In conclusion, multimodal genomic analyses including long read sequencing should be considered in cases where no pathogenic germline variants were detected by conventional genetic testing despite an evident medical or family history of hereditary cancer syndrome. To trigger such a cascade genomic analysis, MGP, which is increasingly used as a front line in genetic testing, should be equipped with a bait design and analytical pipelines to suggest structural variants.

## Data availability statement

The original contributions presented in the study are included in the article/**Supplementary Material**. Further inquiries can be directed to the corresponding author.

## Ethics statement

The research protocol was approved by the Ethics Committee of the National Cancer Center Hospital (Tokyo, Japan) (approval #2013-303). The patients/participants provided their written informed consent to participate in this study. Written informed consent was obtained from the individual(s) for the publication of any potentially identifiable images or data included in this article.

## Author contributions

Conception and design: SO and MH; Study development and methods: SO, MU, WN, TY, YS, and MH; Data collection and analysis: SO, MU, WN, MG, NT, TW, YO, KA, HS, TN, KS, TY, YS, and MH; Manuscript writing: SO, TY, and MH. All authors contributed to the article and approved the submitted version.
