# Supplementary material for: A complex rearrangement between APC and TP63 associated with familial adenomatous polyposis identified by multimodal genomic analysis: a case report
Source: Front Oncol. 2023 Aug 3;13:1205847. doi: 10.3389/fonc.2023.1205847 (PMC10434623; doi:10.3389/fonc.2023.1205847)
Supplement: Supplementary file 1 [file DataSheet_1.pdf]

Supplementary Figure S1

A

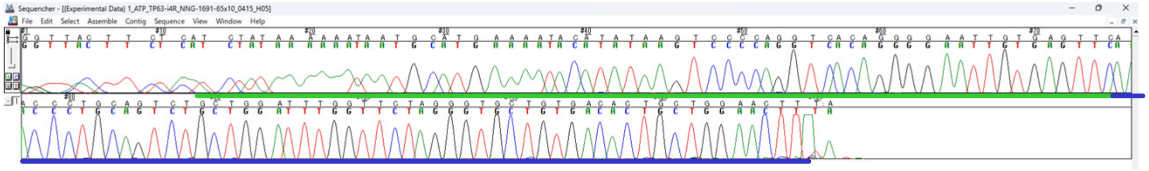

B

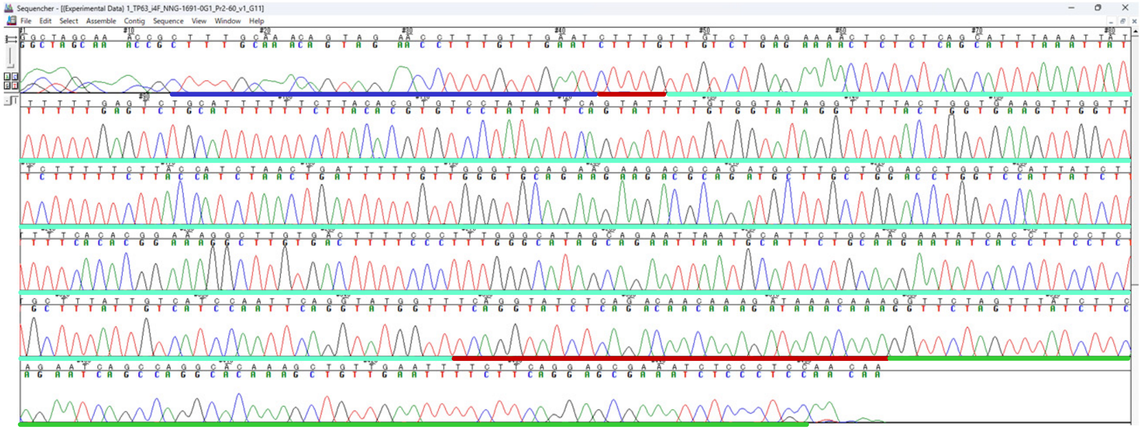

C

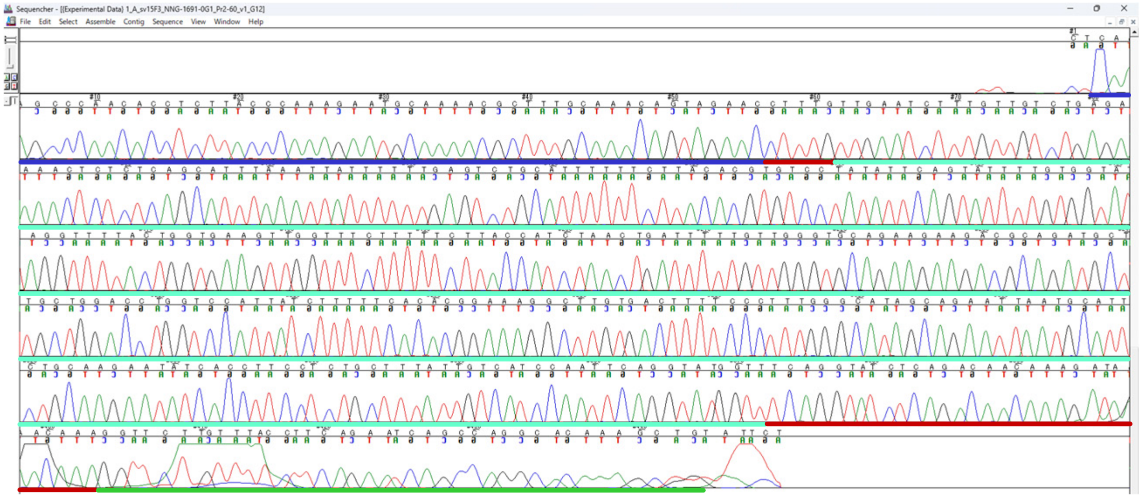

Supplementary Figure S1. Chromatograms around breakpoints in *APC* and *TP63* of the proband. A Genomic sequence by using primer set 1 (Supplementary Table S2), which corresponds to Figure 2C. **B and C** Genomic sequences by using primer set 2 (Supplementary Table S2), which correspond to Figure 2D and E, respectively. Blue bars indicate sequences of *TP63;* red bars, insertion sequences of unknown origin; green bars, sequences of *APC*; light green bars, inversed sequences of *APC*.

Supplementary Figure S2

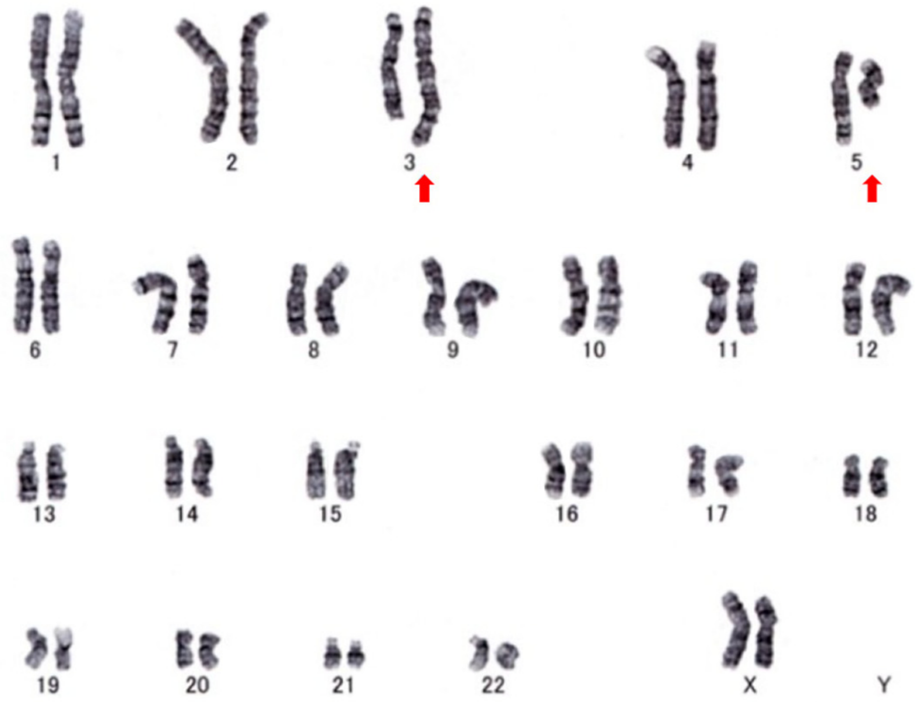

Supplementary Figure S2. Representative G-banded karyotype of the proband. Karyotype: 46,XX,der(3)(3pter→3q12::5q?15 → 5q?22::3q12→3q28::5q22→5qter), der(5)(5pter→5q15::3q28→3qter), based on G-banding and spectral karyotyping. Red arrows indicate derivative chromosomes 3 and 5.

Supplementary Figure S3

A ***APC*** **(5q22.2)**

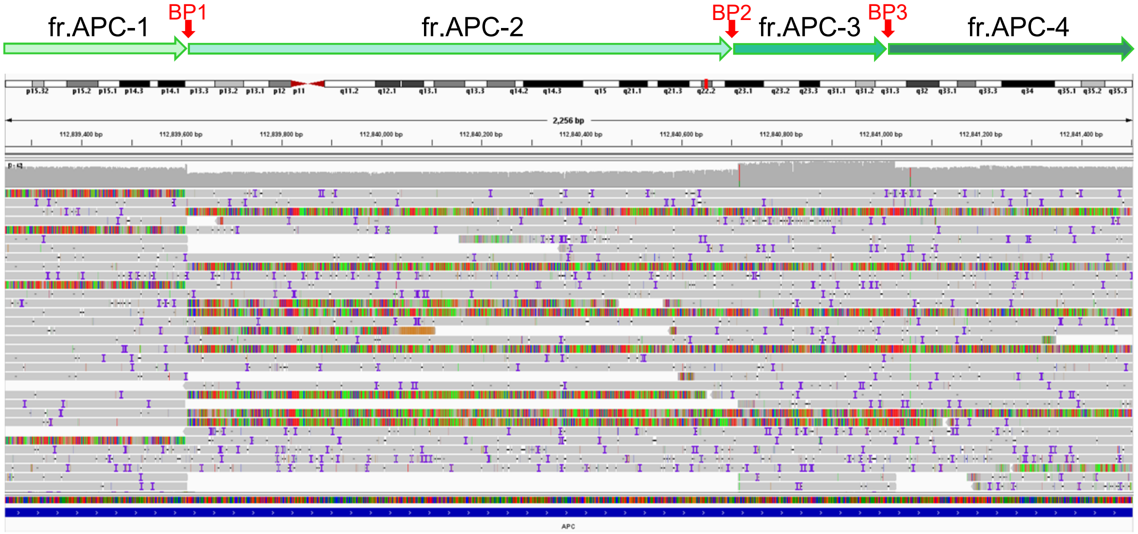

B ***TP63*** **(3q28)**

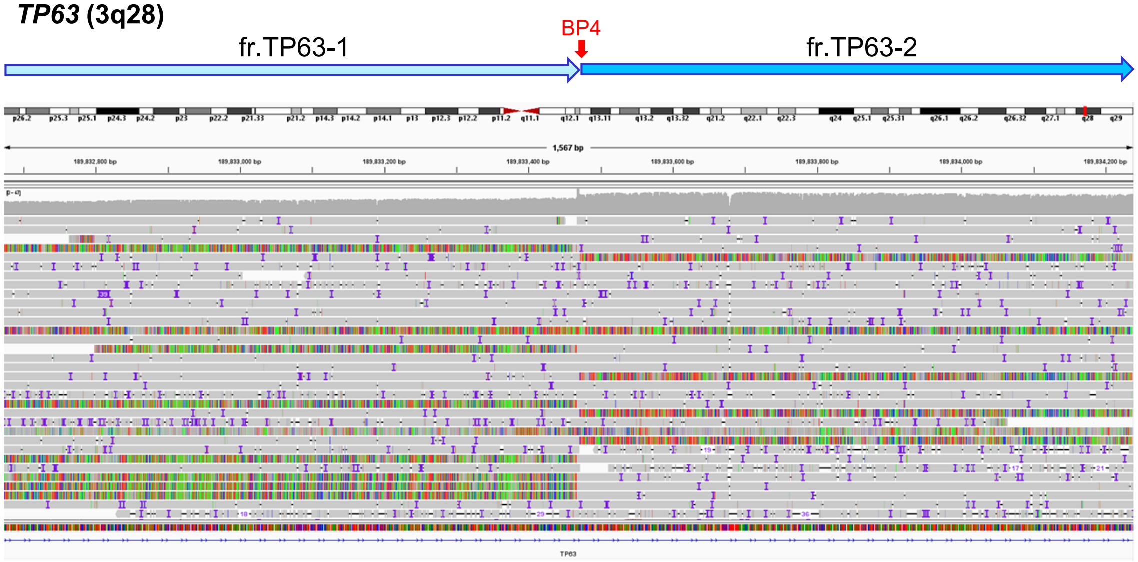

Supplementary Figure S3. Breakpoints in *APC* (A) and *TP63* (B) confirmed by long-read sequencing. Top box of IGV indicates chromosomal positions; middle, mapped read depth; bottom, mapped read sequences. Vertical red arrows indicate 3 (BP1-3) and 1 (BP4) breakpoints in *APC* and *TP63*, respectively, which were presumed by soft-clipped reads and read-depth gaps.

Supplementary Figure S4

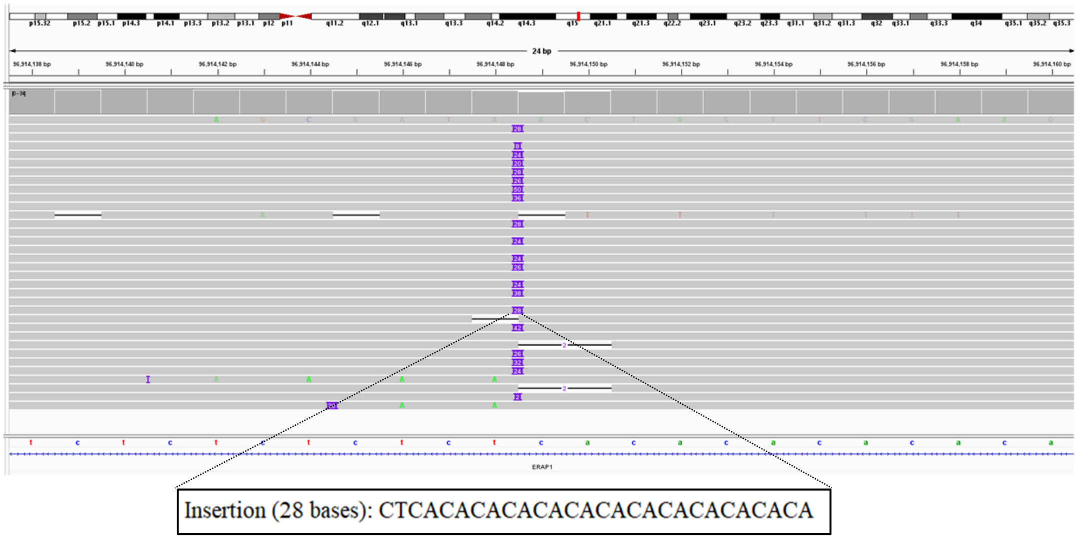

Supplementary Figure S4. Additional structural variant identified by long-read sequencing. Another insertion of simple repeat sequences in a fragment X on der3 was displayed by IGV.
